# Data defining markers of human neural stem cell lineage potential

**DOI:** 10.1016/j.dib.2016.02.030

**Published:** 2016-02-19

**Authors:** Lotta E. Oikari, Rachel K. Okolicsanyi, Lyn R. Griffiths, Larisa M. Haupt

**Affiliations:** Genomics Research Centre, Institute of Health and Biomedical Innovation, Queensland University of Technology, Brisbane, Australia

**Keywords:** Neural stem cells, Neural progenitor cells, Proteoglycans, Lineage, Characterisation

## Abstract

Neural stem cells (NSCs) and neural progenitor cells (NPCs) are self-renewing and multipotent cells, however, NPCs are considered to be more lineage-restricted with a reduced self-renewing capacity. We present data comparing the expression of 21 markers encompassing pluripotency, self-renewal (NSC) as well as neuronal and glial (astrocyte and oligodendrocyte) lineage specification and 28 extracellular proteoglycan (PG) genes and their regulatory enzymes between embryonic stem cell (ESC)-derived human NSCs (hNSC H9 cells, Thermo Fisher) and human cortex-derived normal human NPCs (nhNPCs, Lonza). The data demonstrates expression differences of multiple lineage and proteoglycan-associated genes between hNSC H9 cells and nhNPCs. Data interpretation of markers and proteoglycans defining NSC and neural cell lineage characterisation can be found in “*Cell surface heparan sulfate proteoglycans as novel markers of human neural stem cell fate determination*” (Oikari et al. 2015) [1].

**Specifications** **Table**

**Table:** 

**Subject area**	***Cell biology***
**More specific subject area**	*Human neural stem cell (hNSC) and human neural progenitor cell (hNPC) marker characterisation*
**Type of data**	*Text file, graphs and immunofluorescence images*
**How data was acquired**	*in vitro culture/expansion and phase-contrast fluorescence microscopy data for phenotypic analysis was obtained on an Olympus IX81 inverted fluorescent microscope via Volocity Imaging package; raw Q-PCR data was obtained on* Applied Biosystems 7900HT Fast Real-Time PCR system
**Data format**	*Analysed*
**Experimental factors**	*hNSC H9 and nhNP cells were cultured under basal medium conditions*
**Experimental features**	*hNSC H9 cells (Thermo Fisher) were cultured as a monolayer and nhNP cells (Lonza) were cultured as neurospheres in standard maintenance medium provided by the manufacturer. RNA was harvested and transcribed to cDNA and gene expression of a panel of 49 genes examined by Q-PCR. Specific neural cell lineage markers were further detected through immunofluorescence (IF)*
**Data source location**	*Institute of Health and Biomedical Innovation, Queensland University of Technology, Brisbane, Queensland Australia*
**Data accessibility**	*Data is provided in this article*

• The data provides an extensive panel of markers for better characterisation of human NSCs and NPCs.
• The data demonstrates significant and specific differences in expression of pluripotency, NSC self-renewal and neural cell lineage markers between hNSCs and hNPCs.
• The marker profile data could be used to identify and differentiate between the two cell types to improve their efficacy in research or therapeutic applications.
• The data provides information on the proteoglycan profile of human NSCs and NPCs providing potential new additional markers defining lineage progression of NSCs to NPCs.

## 1 Data

**Fig. 1:**
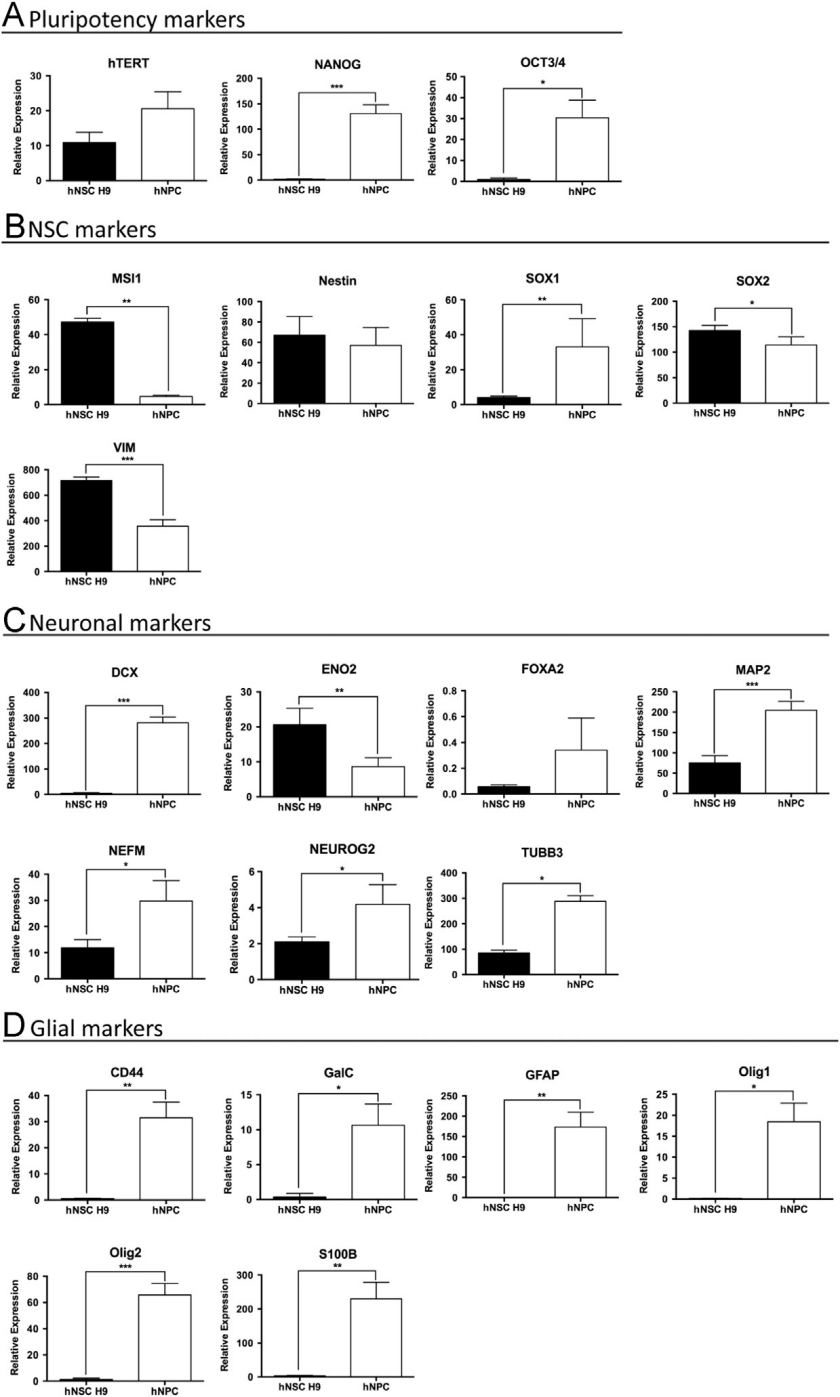
Q-PCR analysis of pluripotency, NSC self-renewal, neuronal and glial lineage marker genes in hNSC H9 cells and nhNPCs. Relative expression in hNSC H9 cells and nhNPCs of: (A) pluripotency markers; (B) NSC self-renewal markers; (C) neuronal lineage defining markers; and (D) glial lineage defining markers. Relative expression normalised to 18 S, error bars=SD, statistical significance: ^⁎^*p*<0.05, ^⁎⁎^*p*<0.01, ^⁎⁎⁎^*p*<0.001.

**Fig. 2:**
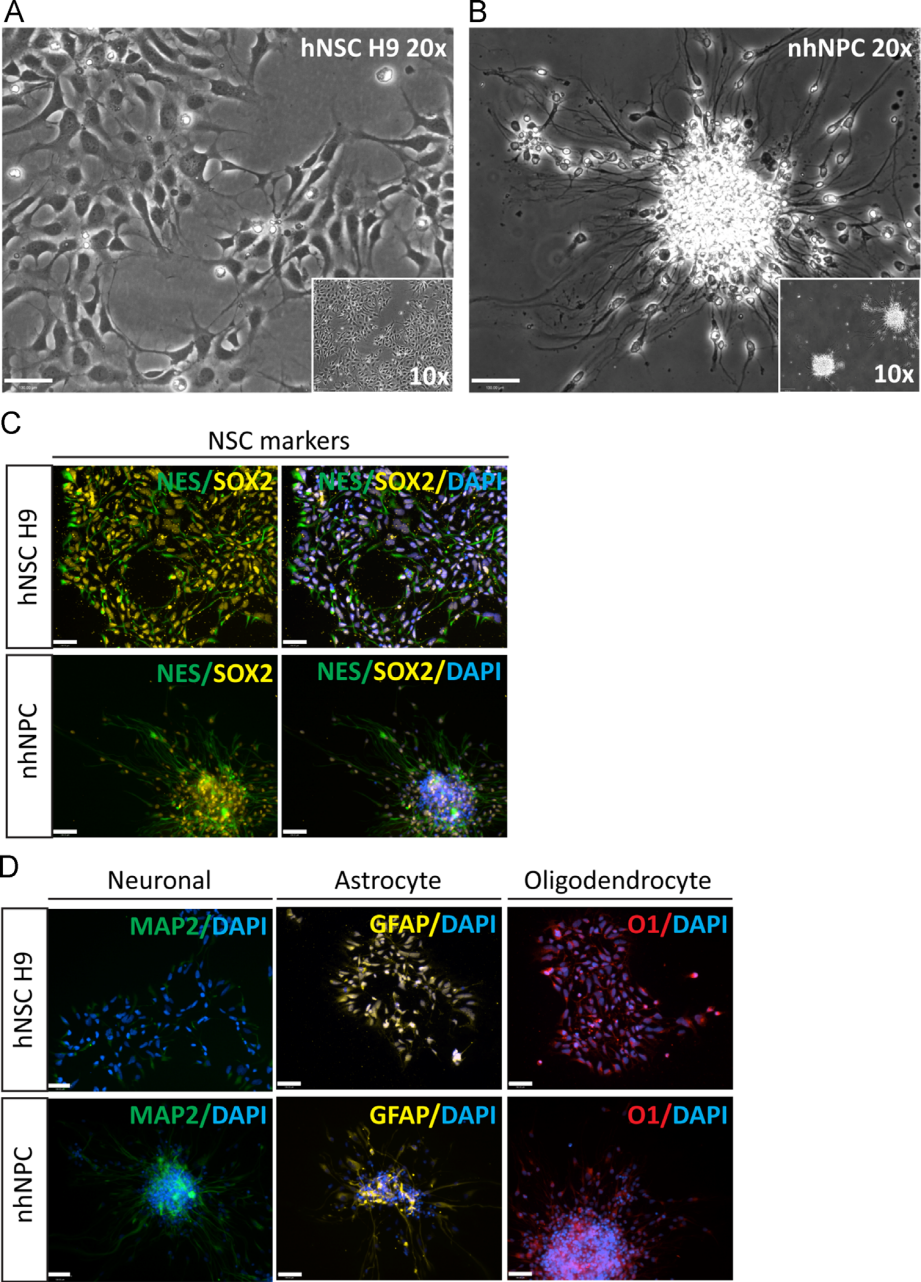
Phenotypic and IF examination of NSC and neural lineage markers in hNSC H9 cells and nhNPCs. Phase-contrast images (20× magnification with 10× magnification inset, scale bar 130 μM) of: (A) hNSC H9 cells at P3 and (B) nhNPCs attached to surface (CC2 chamber slide) during expansion. Immunofluorescence (20× magnification, scale bar 130 μM) in hNSC H9 P3 cells and nhNPCs of stemness and lineage markers: (C) NSC self-renewal markers Nestin (FITC/green) and SOX2 (Cy3/yellow); and (D) neuronal marker MAP2 (FITC/green), astrocyte marker GFAP (Cy3/yellow) and oligodendrocyte marker O1 (AF594/red).

**Fig. 3:**
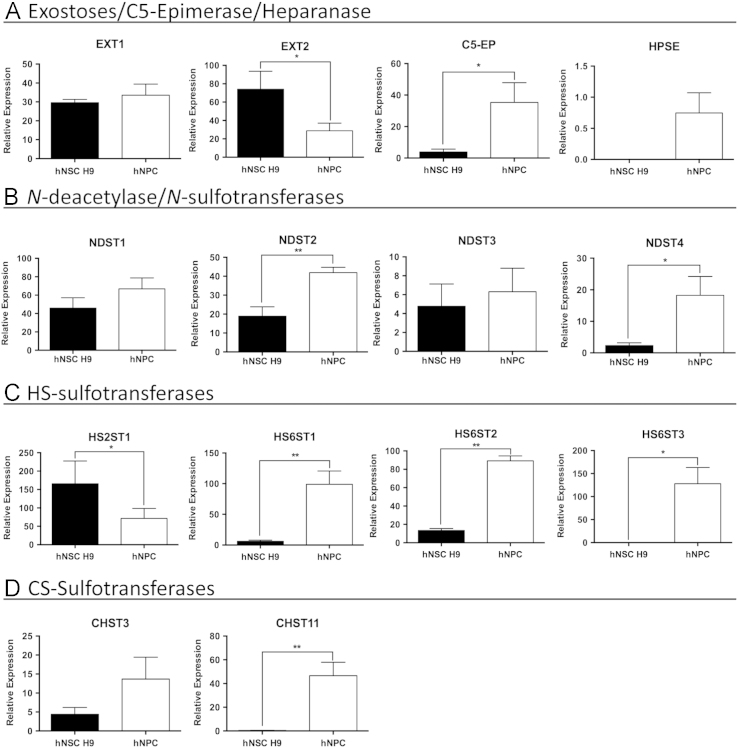
Q-PCR analysis of proteoglycan biosynthesis enzyme gene expression in hNSC H9 cells and nhNPCs. Relative expression in hNSC H9 cells and nhNPCs of: (A) HS chain synthesising and modifying enzymes; (B) HS chain *N*-deacelylating/*N*-sulfating enzymes; (C) HS chain sulfating enzymes; and (D) CS chain sulfating enzymes. Relative expression normalised to 18 S, error bars=SD, statistical significance: ^⁎^*p*<0.05, ^⁎⁎^*p*<0.01, ^⁎⁎⁎^*p*<0.001.

**Fig. 4:**
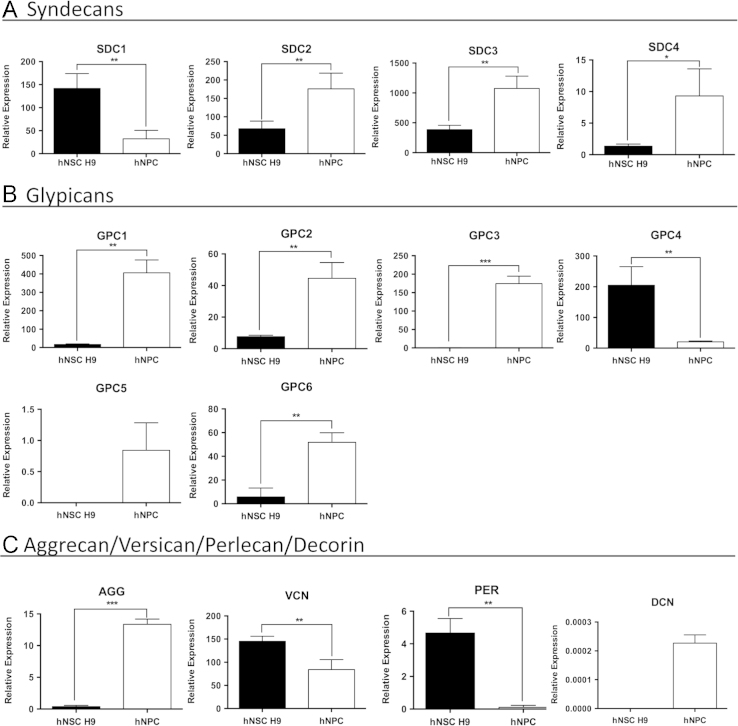
Q-PCR analysis of proteoglycan core protein gene expression in hNSC H9 cells and nhNPCs. Relative expression in hNSC H9 cells and nhNPCs of: cell surface HSPGs (A) syndecans; (B) glypicans; and extracellular CSPGs and HSPGs (C) aggrecan, versican, perlecan and decorin. Relative expression normalised to 18 S, error bars=SD, statistical significance: ^⁎^*p*<0.05, ^⁎⁎^*p*<0.01, ^⁎⁎⁎^*p*<0.001.

We compared the expression of 49 selected genes between human NSCs (hESC-derived hNSC H9 cells, Thermo Fisher) and normal human progenitor cells (nhNPCs, Lonza) following short-term culture under basal growth conditions. Q-PCR data was obtained for pluripotency genes, NSC, neuronal, astrocyte and oligodendrocyte lineage defining genes (*n*=21; Table 1.) (Fig. 1) with several of these markers also detected through immunofluorescence (IF) (Fig. 2) using specific antibodies (Table 3). In addition, Q-PCR data was obtained for 28 heparan and chondroitin sulphate proteoglycan biosynthesis enzymes and core protein genes (Table 2) ubiquitous to the neural niche [1], [2], [3], [4], [5], [6], [7] in hNSC H9 cells and nhNPCs (Fig. 3, Fig. 4). The data presented provides information on self-renewal and multilineage potential as well as proteoglycan expression differences between the two neural stem/progenitor cell types.

## 2 Experimental design, materials and methods

### 2.1 Cell culture

Gibco® human neural stem cells derived from NIH-approved H9 (WA09) embryonic stem cells (hNSC H9 cells) were cultured as a monolayer on Geltrex® coated culture dishes in StemPro® NSC serum-free medium (NSC SFM) containing KnockOUT^™^ DMEM/F-12 supplemented with 2% StemPro® Neural Supplement, 20 ng/ml FGFb and EGF and 2 mM GlutaMAX^™^ (cells and culture reagents obtained from Thermo Fisher). hNSC H9 cells were cultured in p35 (10 cm^2^) dishes with culture medium changed every two days and cells passaged at 90% confluence using TrypLE. hNSC H9 cells were passaged twice and harvested for RNA at passage 3 (P3). Normal Human Neural Progenitor Cells (nhNPCs) isolated from the human brain cortex were cultured as neurospheres in Neural Progenitor Maintenance Bulletkit^™^ medium (NPMM) containing 200 mL of Neural Progenitor Basal Medium supplemented with 0.4 mL rhFGF-B, 0.4 mL rhEGF, 4 mL Neural Survival Factor-1 and 0.4 mL Gentamicin/Amphotericin (cells and culture reagents obtained from Lonza). nhNPC neurosphere cultures were established by defrosting the cell ampule according to the manufacturer’s instructions and dividing the cells into two T75 (75 cm^2^) flasks containing 20 mL of NPMM. hNSC H9 and nhNPCs cultures were maintained in 5% CO_2_ at 37 °C in a humidified atmosphere with phenotype of the cells monitored under an Olympus IX81 inverted phase-contrast microscope.

### 2.2 RNA extraction

RNA was harvested from cultured cells using TRIzol® reagent (Invitrogen) using the Direct-zol^™^ RNA miniprep kit (Zymo Research) according to the manufacturer’s instructions with samples treated in-column with DNase I (Zymo Research). RNA was eluted in RNase-free H_2_O and concentration and quality of RNA determined with a NanoDrop spectrophotometer (Thermo Scientific).

### 2.3 cDNA synthesis

For conversion of RNA into cDNA, 150 ng of RNA was incubated with 200 ng of Random Primer (New England BioLabs) at 65 °C for 10 min in a reaction made up to 20 μl with Milli-Q-H_2_O. Samples were then incubated with 10 U of Transcriptor Reverse Transcriptase (Roche) and 1 mM dNTPs (New England Biolabs), 20 U of RNaseOUT (Invitrogen) in 1x RT reaction buffer in a total reaction volume of 30 μl. For the reverse transcription reaction samples were incubated at 25 °C for 10 min, then at 55 °C for 30 min and finally at 85 °C for 5 min. Concentration and quality of cDNA was measured on a NanoDrop spectrophotometer and cDNA was diluted to 40 ng/mL working concentrations.

### 2.4 Quantitative real-time PCR

Relative gene expression was detected using quantitative real-time PCR (Q-PCR). The 10 μl reaction volume contained 5 μl of SYBR®-Green PCR Master Mix (Promega), 200 ng of forward and reverse primer, 0.1 μl CXR reference dye (Promega) and 120 ng cDNA template. Amplification was monitored on an Applied Biosystems 7900HT Fast Real-Time PCR system with an enzyme activation of 2 min at 50 °C and 3 min at 95 °C followed by 50 cycles of 3 s at 95 °C and 30 s at 60 °C. The cycle threshold (Ct) values were normalised against the endogenous control 18 S (forward primer TTCGAGGCCCTGTAATTGGA, reverse primer GCA GCAACTTAATATACGCTAT) Ct values (Δ*Ct* value) included in each run, and relative gene expression was determined by the ΔΔ*Ct* value (2^(−Δ*Ct*)^). For ease of graphic presentation of relative gene expression, ΔΔ*Ct* values were multiplied by 10^6^. Primer sequences for detected NSC and neural lineage genes are presented in Table 1 and primer sequences for heparan and chondroitin sulphate proteoglycan associated genes are presented in Table 2.

#### 2.4.1 Immunofluorescence (IF)

Expression of selected NSC and neural lineage marker proteins were detected via IF using an Olympus IX81 inverted phase-contrast fluorescent microscope and images acquired using Volocity software (Perkin-Elmer) on a Hamamatsu Orca camera. For imaging, cells were plated on 8-well CC2-coated chamber slides (Lab-Tek) at 20–30×10^4^ cells/well and cells were cultured for 3–4 days before fixing and staining. Briefly, culture medium was removed, cells rinsed with 1× PBS with Ca^2+^ and Mg^2+^ and fixed with 4% paraformaldehyde. After this cells were blocked (5% Donkey serum, 1% BSA in PBS with or without 0.1% Triton-X to allow permeabilisation) and primary antibodies were incubated overnight at 4 °C. Isotype control antibodies were used as a negative control. After 24 h incubation, primary antibodies were removed, cells rinsed with 1× PBS with Ca^2+^ and Mg^2+^ and cells incubated with secondary antibodies for 2 h at room temperature. Finally, cells were rinsed with 1× PBS with Ca^2+^ and Mg^2+^ and slides mounted with DAPI (ab104139, Abcam). Antibodies and dilutions used are presented in Table 3.

#### 2.4.2 Statistical analysis

For Q-PCR analysis each gene was detected in quadruplicate per sample. Paired *t*-test was used to determine statistical significance and defined as * *p*<0.5, ** *p*<0.01 and *** *p*<0.001. Error bars represent SD.

## Figures and Tables

**Table 1:** Primer sequences of NSC and neural lineage related genes.

**Gene**	**Symbol**	**Forward primer**	**Reverse primer**	**RefSeq**	**Ref**
**CD44**	CD44	AGCAACTGAGACAGCAACCA	AGACGTACCAGCCATTTGTGT	NM_000610.3	
**Doublecortin**	Dcx	TATGCGCCGAAGCAAGTCTC	TACAGGTCCTTGTGCTTCCG	NM_178152.2	
**Enolase 2**	ENO2	TGCACAGGCCAGATCAAGAC	ACAGCACACTGGGATTACGG	NM_001975.2	
**Forkhead box A2**	FOXA2	CTGGTCGTTTGTTGTGGCTG	GGAGGAGTAGCCCTCGG	NM_021784.4	
**Galactosylceramidase**	GalC	GCCAAGCGTTACCATGATTT	TTTCACTCGCTGGAGACCTT	NM_001201402.1	[8]
**Glial fibrillary acidic protein**	GFAP	GAGGTTGAGAGGGACAATCTGG	GTGGCTTCATCTGCTTCCTGTC	NM_002055.4	
**Human telomerase**	hTERT	GACGTGGAAGATGAGCGTG	GACGACGTACACACTCATC	NM_001193376.1 NM_198253.2	[9]
**Microtubule associated protein 2**	MAP2	GACTGCAGCTCTGCCTTTAG	AAGTAAATCTTCCTCCACTGTGAC	NM_002374.3	
**Mushahi I**	MSI I	TGACCAAGAGATCCAGGGGT	CGATTGCGCCAGCACTTTAT	NM_002442.3	
**Nanog homeobox**	NANOG	ACCTCAGCTACAAACAGGTGAA	AAAGGCTGGGGTAGGTAGGT	NM_024865.2	
**Nestin**	NES	CTCAGCTTTCAGGACCCCAA	GTCTCAAGGGTAGCAGGCAA	NM_006617.1	
**Neurofilament M**	NEFM	TGCAGTCCAAGAGCATCGAG	GGATGGTGTCCTGGTAGCTG	NM_005382.2	
**Neurogenin 2**	NEUROG2	AGAGCCAACTAAGATGTTCGTCA	CGATCCGAGCAGCACTAACA	NM_024019.3	
**Oligodendrocyte transcription factor 1**	OLIG1	GTCATCCTGCCCTACTCAGC	CTGCCCAGCAGTAGGATGTAG	NM_138983.2	[8]
**Oligodendrocyte transcription factor 2**	OLIG2	GACAAGCTAGGAGGCAGTGG	CGGCTCTGTCATTTGCTTCT	NM_005806.3	[8]
**POU Class 5 homeobox 1 (OCT3/4)**	OCT3/4	ATCTTCAGGAGATATGCAAAGCAGA	TGATCTGCTGCAGTGTGGGT	NM_002701.4	
**SRY box 1**	SOX1	CAACCAGGACCGGGTCAAAC	CCTCGGACATGACCTTCCAC	NM_005986.2	
**SRY box 2**	SOX2	CCACCTACAGCATGTCCTACTCG	GGGAGGAAGAGGTAACCACAGG	NM_003106.3	[10]
**S100 Calcium binding protein B**	S100B	TTCTGGAAGGGAGGGAGACA	CTCCTGCTCTTTGATTTCCTCT	NM_006272.2	
**Vimentin**	VIM	GGACCAGCTAACCAACGACAAA	CGCATTGTCAACATCCTGTCTG	NM_003380.3	
**βIII tubulin**	TUBB3	GGCCAAGTTCTGGGAAGTCAT	CTCGAGGCACGTACTTGTGA	NM_06086.3	

**Table 2:** Primer sequences of proteoglycan associated genes.

**Gene**	**Symbol**	**Forward primer**	**Reverse primer**	**RefSeq**	**Ref**
**Aggrecan**	AGG	TGCATTCCACGAAGCTAACCTT	CGCCTCGCCTTCTTGAAATGT	NM_001135	
**C5-Epimerase**	C5-EP	AGCTGTCAAGCCAACCAAAATAA	CTTACTAGCCAATCACTAGCAGCAA	AY635582	
**carbohydrate (chondroitin 6) sulfotransferase 3**	CHST3	GGTTTTTGTGGTGATAGTTTTTGTCTT	GCTGGGTCGGTGCTGTTG	NM_004273	
**carbohydrate (chondroitin 4) sulfotransferase 11**	CHST11	CTGCTGGAAGTGATGAGGATGA	GATGTCCACACCAAAGGGATTC	NM_018413	
**Decorin**	DCN	TCCTGATGACCGCGACTT	GAGTTGTGTCAGGGGGAAGA	NM_001920.3	
**Exostose 1**	EXT1	TGACAGAGACAACACCGAGTATGA	GCAAAGCCTCCAGGAATCTGAAG	NM_000127.2	
**Exostose 2**	EXT2	CAGTCAATTAAAGCCATTGCCCTG	GGGATCAGCGGGAGGAAGAG	NM_000401	
**Glypican 1**	GPC1	GGACATCACCAAGCCGGACAT	GTCCACGTCGTTGCCGTTGT	NM_002081	
**Glypican 2**	GPC2	TGATCAGCCCCAACAGAGAAA	CCACTTCCAACTTCCTTCAAACC	NM_152742	
**Glypican 3**	GPC3	GATACAGCCAAAAGGCAGCAA	GCCCTTCATTTTCAGCTCATG	NM_004484.	
**Glypican 4**	GPC4	GGTGAACTCCCAGTACCACTTTACA	GCTTCAGCTGCTCCGTATACTTG	NM_001448	
**Glypican 5**	GPC5	GCTCACCTCAATGGACAAAAATT	GTTGGCAAGCGTCTCTTCACT	NM_004466	
**Glypican 6**	GPC6	CAGCCTGTGTTAAGCTGAGGTTT	GATGTGTGTGCGTGGAGGTATGT	NM_005708.	
**Heparanase**	HPSE	TCACCATTGACGCCAACCT	CTTTGCAGAACCCAGGAGGAT	NM_006665.5	
**Heparan sulphate 2-*O* sulfotransferase 1**	HS2ST1	TCCCGCTCGAAGCTAGAAAG	CGAGGGCCATCCATTGTATG	NM_012262	
**Heparan sulphate 6-*O* sulfotransferase 1**	HS6ST1	AGCGGACGTTCAACCTCAAGT	GCGTAGTCGTACAGCTGCATGT	NM_004807	
**Heparan sulphate 6-*O* sulfotransferase 2**	HS6ST2	TCTGGAAAGTGCCAAGTCAAATC	ATGGCGAAATAAAGTTCATGTTGAA	NM_147175	
**Heparan sulphate 6-*O* sulfotransferase 3**	HS6ST3	ACATCACGCGGGCTTCTAACGT	GGCGGTCCCTCTGGTGCTCTA	NM_153456	
***N*-deacetylase/*N*-sulfotransferase 1**	NDST1	TGGTCTTGGATGGCAAACTG	CGCCAAGGTTTTGTGGTAGTC	NM_001543	
***N*-deacetylase/*N*-sulfotransferase 2**	NDST2	CCTATTTGAAAAAAGTGCCACCTACT	GCAGGGTTGGTGAGCACTGT	NM_003635	
***N*-deacetylase/*N*-sulfotransferase 3**	NDST3	ACCCTTCAGACCGAGCATACTC	CCCGGGACCAAACATCTCTT	NM_004784	
***N*-deacetylase/*N*-sulfotransferase 4**	NDST4	ATAAAGCCAATGAGAACAGCTTACC	GGTAATATGCAGCAAAGGAGATTGA	NM_022569	
**Perlecan**	PER	TGGACACATTCGTACCTTTCTGA	CCTCGGACACCTCTCGAAACT	NM_005529	
**Syndecan 1**	SDC1	CTGGGCTGGAATCAGGAATATTT	CCCATTGGATTAAGTAGAGTTTTGC	BC008765.2	
**Syndecan 2**	SDC2	AGCTGACAACATCTCGACCACTT	GCGTCGTGGTTTCCACTTTT	NM_002998.3	
**Syndecan 3**	SDC3	CTTGGTCACACTGCTCATCTATCG	GCATAGAACTCCTCCTGCTTGTC	AF248634	
**Syndecan 4**	SDC4	CCACGTTTCTAGAGGCGTCACT	CTGTCCAACAGATGGACATGCT	BC030805.1	
**Versican**	VCN	TGGAATGATGTTCCCTGCAA	AAGGTCTTGGCATTTTCTACAACAG	NM_004385.4	

**Table 3:** Antibodies used for immunofluorescence.

***Primary antibodies***	***Dilution***	***Company (Cat#)***
Anti-Nestin (Mouse IgG)	1:200	Abcam (ab22035)
Anti-SOX2 (Rabbit IgG)	1:1000	Millipore (2003600)
Anti-MAP2 (Mouse IgG)	1:200	Abcam (ab36447)
Anti-GFAP (Rabbit IgG)	1:250	Abcam (ab7260)
Anti-O1 (Mouse IgM)	1:500	Abcam (ab34164)
***Isotype Controls***	***Dilution***	***Company (Cat#)***
Mouse IgG	1:250	Millipore (PP54-100UG)
Rabbit IgG	1:250	Millipore (PP64-100UG)
Mouse IgM	1:500	Millipore (2003599)
***Secondary antibodies***	**Dilution**	**Company (Cat#)**
Donkey Anti-Mouse IgG (FITC, green)	1:250	Millipore (AP192F)
Donkey Anti-Rabbit (H+L) (Cy3, yellow)	1:250	Millipore (AP182C)
Donkey Anti-Mouse IgM (AlexaFluor 594, red)	1:500	Jackson Immunoresearch (#715-585-020)
