# Supplementary material for: Data defining markers of human neural stem cell lineage potential
Source: Data Brief. 2016 Feb 19;7:206–15. doi: 10.1016/j.dib.2016.02.030 (PMC4773572; doi:10.1016/j.dib.2016.02.030)

AUTHOR DECLARATION TEMPLATE

On behalf of all the authors, as the Corresponding Author, I wish to confirm that there are no known conflicts of interest associated with this publication and there has been no significant financial support for this work that could have influenced its outcome.

I further confirm on behalf of all Authors that the manuscript has been read and approved by all named authors and that there are no other persons who satisfied the criteria for authorship but are not listed. I can confirm that the order of authors listed in the manuscript has been approved by all authors.

I confirm that we have given due consideration to the protection of intellectual property associated with this work and that there are no impediments to publication, including the timing of publication, with respect to intellectual property. In so doing we confirm that we have followed the regulations of our institutions concerning intellectual property.

All authors have acknowledged that as the Corresponding Author (Dr Larisa M Haupt), I am the sole contact for the Editorial process (including Editorial Manager and direct communications with the office). This includes the responsibility of communicating with the other authors about progress, submissions of revisions and final approval of proofs. I further confirm that all authors have provided me with a current, correct email address for communication regarding the manuscript.

Please address any correspondence concerning this publication to:
Dr Larisa M. Haupt
Genomics Research Centre,
Institute of Health and Biomedical Innovation
Queensland University of Technology
Queensland 4059 Australia
Email: larisa.haupt@qut.edu.au

We thank you in advance for your consideration and await your response.

Yours Sincerely

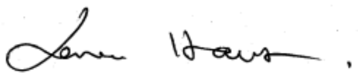

Supplement: Supplementary file 1 — Supplementary material [file mmc1.pdf]
